# Traumatism of the Hip, Simulating Thyroid Dislocation

**Published:** 1889-11

**Authors:** T. H. Myers

**Affiliations:** New York


### TRAUMATISM OF THE HIP, SIMULATING THYROID DISLOCATION.

---

By T. H. MYERS, New York.

---

E. A., aged nine years, was first seen July 29th, 1889, and gave the following history:

She was playing, and fell on the sidewalk three and a half weeks ago, striking on her left hip. She immediately walked to the house, with marked limp, but suffered almost no pain. Her father says that the leg was then in exactly the same position as at present. Since the evening of the fall the patient says she has had no pain. Her sleep has not been disturbed and she has been playing about every day, but the deformity persisted. Dr. W. S. Carr, who attended her a week after the accident, writes me: "There never was any swelling, and but very little tenderness over the greater trochanter. When she stepped, the foot and knee were slightly everted."

EXAMINATION. In standing patient holds left thigh advanced, with slight knee flexion, and foot in equinus, with 45° of outward rotation of thigh. In supine position there is flexion at the hip of 160°, and abduction of 30°. When legs are approximated, the left is apparently 1¼ inches longer than the right. There is, however, no *real* difference. There is a marked depression over trochanter major, and the trochanter is ⅜ of an inch above Nelaton's; on flexing both thighs to 90°, left knee is one inch higher than the right; but *all these facts seem due to the abduction.* Adduction and extension are impossible. Rotation, abduction and flexion are allowed to a considerable extent and when limited, there is not the quick, hesitating spasm characteristic of the second stage of morbus coxarius, but rather the firm resistance of a dislocation, or an ankylosis.

The whole history, except the absence of pain, suggested thyroid dislocation, and in respect to pain, I remembered that Dr. L. A. Stimson had lately mentioned cases of this dislocation where pain was not complained of, the patients seeking advice because they "could not bring the legs together."

Repeated examinations were made for the head of the femur, but no displacement could be found. There was slight thickening in the inguinal region but nothing else, and as the patient was not fat, and was examined once under ether, I feel sure that there was no dislocation of the femur.

*Under ether the flexion and abduction disappeared*, but still abduction and extension were restricted. There was no crepitus elicited, nor any separation of the trochanter major. The spasm returned after the ether passed off, but was less marked. The parts about the joint presented the same appearance as before. The day after the examination there was a little pain in the joint, but by the following day it had disappeared, and I found the flexion, abduction and rotation had disappeared also. All the joint motions were very free except extension. There was still some fullness in inguinal region, and the gluteal crease was shallow.

During the next three weeks there was once or twice a little pain and stiffness in the joint. The long traction splint was now applied, and four weeks later I made the following note: "No shortening, no atrophy, motion free and smooth in all directions to full extent. Complains of slight pain in hip at limits of flexion on *both* sides. Spine and pelvis normal. Pressure over trochanter causes a little superficial pain there. Patient allowed up without support. The child had been perfectly well before the accident, and her family history is good.

The nature of the accident and the onset of the symptoms negative a diagnosis of morbus coxarius. The examination excluded thyroid dislocation and peri-articular affections; acute synovitis should have given pain.

In view of these facts, and because we know that involuntary muscular spasm is an indication of bone disease, and especially of that part of the bone lying immediately below the articular cartilages; and for the further reason that pathological reports show that a separation to a greater or less degree of the cotyloid ligament and the bone, sometimes occurs in cases of dislocation proper, even where the pressure is relieved by the rupture of the capsule. I would suggest that the lesion in my case was probably a partial separation of this kind.

The literature of traumatism of the hip without fracture or dislocation is so meagre that I present this case rather fully, feeling especially interested in the questions of pathology and prognosis.

The patient has now been without protection for but four days, and I do not consider the case cured, though all motions are free and smooth to the extreme normal limit. There is, however, when both knees are brought in contact with the chest wall a little more tenderness in left than in right groin, and there is still, on very close observation, a slight tendency to favor the left leg in walking and running.

